# Best practices for extracorporeal shockwave therapy in musculoskeletal medicine: Clinical application and training consideration

**DOI:** 10.1002/pmrj.12790

**Published:** 2022-04-14

**Authors:** Adam S. Tenforde, Haylee E. Borgstrom, Stephanie DeLuca, Molly McCormack, Mani Singh, Jennifer Soo Hoo, Phillip H. Yun

**Affiliations:** ^1^ Department of Physical Medicine and Rehabilitation Spaulding Rehabilitation Hospital/Harvard Medical School Charlestown Massachusetts USA; ^2^ Department of Rehabilitation Medicine New York‐Presbyterian (Columbia/Cornell) New York New York USA; ^3^ Department of Rehabilitation Medicine Weill Cornell Medicine New York New York USA; ^4^ Department of Medicine Massachusetts General Hospital/Harvard Medical School Boston Massachusetts USA

## INTRODUCTION

Extracorporeal shockwave therapy (ESWT) is used in a variety of clinical applications including the management of musculoskeletal conditions. Initial use of ESWT in clinical practice was in urological application of lithotripsy in the early 1980s and subsequently expanded to musculoskeletal conditions.^1^ Physiologic effects of shockwaves have been widely investigated with observations that different energy forms can have effects on the musculoskeletal system, reducing pain and facilitating tissue healing. Beneficial effects have been shown in the management of musculoskeletal disorders including calcific tendinopathy, plantar fasciitis, and lateral epicondylosis.^2^, ^3^, ^4^

Expanded clinical use of ESWT for the treatment of musculoskeletal conditions has been observed. The different forms of ESWT and treatment protocols contribute to variable application in clinical practice. Reported differences in treatment approach have contributed to the conflicting evidence in the current literature for shockwave therapy. This includes differences in energy flux density (EFD), number of impulses, type of ESWT and device (focused vs. radial), frequency and number of treatment sessions, and use of analgesia during application.^5^ This practice management outlines recommendations for adoption and best use of ESWT in outpatient musculoskeletal clinics. We review the science and proposed mechanisms of action for shockwave to treat musculoskeletal injuries, clinical pearls on patient counseling and application of treatment, suggested benchmarks for education of trainees, and future directions for research to determine individualized protocols across conditions and/or patient populations.

## FORMS OF EXTRACORPOREAL SHOCKWAVE THERAPY

Shockwaves are a form of energy that has biological effects described at the cellular, tissue, and organ level. ESWT is currently delivered through two primary forms: focused shockwave therapy (F‐SWT) and radial shockwave therapy (R‐SWT). F‐SWT generates an initially wide field of pressure waves that become focused and converge at a particular depth (the focus) within the body. Three main forms of F‐SWT are used in commercial devices available for clinical use: electrohydraulic, electromagnetic, and piezoelectric.^6^ In contrast, R‐SWT generates pressure waves that exert maximal pressure at the applicator and attenuate as waves travel to deeper structures.^1^ These outward directed pressure waves are generated via different mechanisms compared to F‐SWT.^1^, ^6^ Compared with F‐SWT, the pressure waves generated in R‐SWT reach lower speeds, generate lower peak pressures, and may not generate a “true” shockwave leading some to suggest alternative terminology of radial pressure wave therapy.^7^ The physical effects of ESWT have been shown to relate to the energy per unit area (EFD, mJ/mm^2^) or maximal positive pressure (bar), which can be thought of as the ESWT dosage.^1^

Understanding these concepts may explain mechanisms for use of different devices for a given condition. In general, R‐SWT has a more superficial effect, whereas F‐SWT has the capacity to achieve effects further from the site of application.^7^ Multiple head‐to‐head studies have compared the effectiveness of R‐SWT versus F‐SWT for conditions including tendinopathy and spasticity.^8^, ^9^ The results suggest that both forms of shockwave therapy are appropriate, but differences in mechanistic effects may result in different outcomes for a given condition.^9^, ^10^, ^11^

## PROPOSED MECHANISMS OF ACTION

Although clinical evidence has suggested that ESWT is effective in treating musculoskeletal conditions, the precise mechanisms remain unknown. Cellular mechanotransduction, or mechanotherapy, explains how mechanical stimuli lead to cellular migration, proliferation, differentiation, or apoptosis.^1^ Higher levels of energy may result in disruptive, shear stresses rather than serving as a mechanical stimulus.^7^ Pain modulation may induce cellular changes explained by the principle of hyperstimulation analgesia.^12^ See Table 1 for full proposed mechanisms of actions of shockwave therapy, with references to a review^5^ and the International Society for Medical Shockwave Treatment^14^ where readers can further identify primary sources of interest that are not included due to limitations in references. Similar reference to selected literature on specific postulated mechanisms of actions for tendons, bone disorders, joint pathology, and spasticity are described in Table 2.

**TABLE 1:** Proposed cellular mechanisms of action for extracorporeal shockwave therapy

Increased collagen synthesis	Enhanced fibroblast proliferation (increased transforming growth factor beta [TGF‐β]) and upregulation of collagen I and III^5^, ^13^
	Regulation of scleraxis, tenomodulin^13^
Cellular proliferation and wound healing	Upregulation of tendon‐derived tenocytes^13^
	Increased ATP release and downstream extracellular signal‐regulated kinase activation^14^
	Enhancement of osteogenesis^5^
	IL‐6 and IL‐8 mediated tendon remodeling^5^
Pain reduction	Gate‐control theory^5^
	Modifies substance P release^14^
	Decreased calcitonin‐gene‐related peptide^14^
Neovascularization	Induction of TGF‐βI and insulin‐like growth factor I^5^
Decrease in soft tissue calcifications^5^	
Decrease in inflammation^5^	

**TABLE 2:** Specific postulated mechanisms of action of extracorporeal shockwave therapy

Pathology	Mechanism of action
Tendons^1^, ^13^	Decreased edema and inflammatory cell infiltration within tendons Tissue regeneration via conversion of mechanical stimulation to biochemical signal Increase transforming growth factor beta‐1 and insulin‐like growth factor I levels to stimulate tenocyte and collagen proliferation (important in healing) Scleraxis upregulation (promotes tendon growth and development) Proliferation of anti‐inflammatory cytokines Increased proliferation and migration of tendon‐derived tenocytes Decreased metalloproteinase expression (enzymes that can degrade collagen) Reduction of inflammatory interleukins
Bones^15^	Protein upregulation may enhance angiogenesis and neovascularization of the bone Osteogenesis and bone remodeling by release of growth factors Bone morphogenic protein 2 Vascular endothelial growth factor Promotion of periosteal bone formation Decreased osteoclast activity Increased osteoblast activity
Joints (knee)^16^	Decreased inflammation Decreased edema Improvements in subchondral bone architecture Increased chondrocyte activity (cartilaginous repair)
Spasticity^17^	Decreased spasticity at the level of the muscle and neuromuscular junction Reduced rigidity of connective tissues (muscle level) Stimulate synthesis of nitric oxide Neuromuscular junction formation Neovascularization

## CLINICAL APPLICATIONS AND TREATMENT PROTOCOLS FOR SHOCKWAVE THERAPY

### Treatment parameters

Clinical application may include a combination of instrumental parameters to better target a particular pathology (Table 3).^7^ ESWT can be separated into three energy categories depending on the EFD. The exact cutoffs between these levels vary in the literature, but general ranges are as follows: low (<0.08–10 mJ/mm^2^), medium (0.08–0.28 mJ/mm^2^), and high (>0.29–0.60 mJ/mm^2^).^18^ There is some evidence that higher energy levels provide more benefit in the treatment of calcific tendinopathy and disorders of bone, whereas low and medium energy levels may promote healing in tendinopathies and spasticity.^7^

**TABLE 3:** Extracorporeal shockwave therapy treatment parameters

Parameter/Variable	Description or unit
Depth of focus	Dependent on targeted pathology
Shockwave device	Multiple types on market
Time interval between treatments	Typically 1 week
Total number of treatments	Typically 3–5
Local anesthesia	Recommend none
Image guidance	Recommend clinical focusing
Type of shockwave therapy	Focused, radial, combined
Total number of impulses per treatment	Pulses
Impulse frequency	Number of shockwaves applied per second
Maximal positive pressure	Maximal positive pressure reached during treatment, measured in bar
Energy flux density (EFD)	Amount of energy/surface area, measured in mJ/mm^2^
Total energy dose (TED)	EFD x total number of impulses = TED

### Clinical application in tendinopathies, fasciopathy, and soft tissue pathologies

Treatment of chronic tendinopathies and plantar fasciitis has been among the most frequently studied applications of ESWT. Both F‐SWT and R‐SWT demonstrated clinical efficacy.^9^, ^10^, ^19^, ^20^ In practice, combining forms of F‐SWT and R‐SWT (C‐SWT) may be an effective treatment option to target different anatomic structures for a given musculoskeletal condition.^21^, ^22^

We recommend that clinical focusing without anesthetics be used in the application of shockwave.^23^ The application of ESWT is based on clinical focusing, described as treatment over areas of maximal pain, which should be used to maximize outcomes and guide application to primary and secondary sites of injury.^23^ For example, treatment of the muscle‐tendon‐bone unit may address soft tissue impairments compared to application of ESWT at the primary site of tendon pathology. For example, mid‐portion Achilles tendinopathy treatment should include direct application over the tendon and exploration of the gastrocnemius, soleus, myotendinous junction, and the enthesis of the Achilles tendon‐calcaneal junction to identify additional sites of injury. Although treatment can be painful and should be accounted for when applying ESWT and counseling patients, it is helpful to understand that there are no established upper limits in total application (unlike concerns regarding corticosteroid, botulinum toxin, or other medication application in treatment of musculoskeletal injury or spasticity management). Therefore, identifying and treating secondary sites of injury can also be performed during a treatment session. In the example of Achilles tendinopathy, treatment of coexisting posterior tibial tendinopathy or secondary plantar fasciitis may optimize function.

### Clinical application for bone stress injuries, delayed union, and avascular necrosis

Documented use of ESWT in the treatment of bone conditions includes treatment of delayed healing of stress fractures, nonunion fractures, and osteonecrosis and avascular necrosis of the femoral head.^15^ When using ESWT, high energy application is required to treat bone as the mechanism to facilitate bone remodeling requires upregulation of localized nitrous oxide to promote angiogenesis.^20^, ^24^

Clinically, the use of F‐SWT to achieve higher EFD to target bone‐related conditions can be accomplished through combination of clinical focusing and existing imaging (e.g., x‐ray, computed tomography, or magnetic resonance imaging). Knowledge of the anatomy and neurovascular structures is important when applying F‐SWT, and ultrasound can be used to visualize structures that are difficult to localize.^15^ Clinical focusing, described as treatment over areas of maximal pain, is used to maximize outcomes and guide application.

### Clinical applications and protocols: joints

ESWT in the treatment of joint pathology is complex, as multiple anatomic structures are targeted simultaneously. Animal models have illustrated the positive effects of F‐SWT on subchondral bone development and neovascularization.^25^ F‐SWT may have a greater impact on ossification, whereas R‐SWT may preferentially target surrounding soft tissues (such as articular cartilage).^16^, ^25^ R‐SWT has been theorized to positively affect soft tissue healing in joints given the success of R‐SWT in other soft‐tissue pathologies.^19^ Given the presence of multiple anatomic structures within joints as well as the difficulty to preferentially target individual structures, C‐SWT may be a more appropriate treatment for joint pathology although research would be helpful to quantify how this affects outcomes.

### Clinical applications in management of spasticity

In the past decade, ESWT has gained traction as a potential treatment for spasticity. Multiple randomized control trials have concluded that ESWT is beneficial in reducing spasticity, reducing pain, improving range of motion, and improving function in conditions including cerebral palsy and stroke with both upper and lower limb spasticity.^9^, ^26^, ^27^ Etiologies studied include cerebral palsy, stroke, and spinal cord injury with both upper and lower limb spasticity.^9^ R‐SWT at lower energy levels has been frequently employed in the management of spasticity as seen in the current literature.^9^, ^17^, ^26^, ^27^ However, similar to other studies on tendinopathies, there are still significant differences between shockwave devices, frequency, and numbers of sessions between research studies.

## CLINICAL PRACTICE PEARLS: EDUCATION, ERGONOMICS, AND THE USE OF TECHNICIANS

### Education

According to Accreditation Council for Graduate Medical Education (ACGME) and American Board of Physical Medicine and Rehabilitation (ABPMR) requirements, graduating physical medicine and rehabilitation (PM&R) residents must meet specific milestones for core competencies including procedural skills and knowledge.^28^ Although specific guidelines have not been established, we propose residency and fellowship training should include education on ESWT and teach the procedure when feasible. We present a framework for an ESWT curriculum based on existing curricular standards, milestones, and competencies adapted from musculoskeletal ultrasound curricula supported by ACGME, ABPMR, and American Medical Society for Sports Medicine.^28^

ESWT core curriculum should include both didactic and clinical components (Table 4). Didactic components may include a combination of formal lecture(s) and hands‐on teaching sessions with demonstration of ESWT by senior residents, fellows, and/or faculty followed by supervised trainee practice with real‐time feedback. Clinical components include a rotation orientation with overview of procedural milestones and competencies, review of safety protocols, ergonomics, techniques, documentation, direct observation of faculty performing informed consent, ESWT procedure, supervised performance of ESWT based on the resident's current milestone achievement, formal feedback and prompt debriefing sessions immediately post procedure, and resident competency assessment.

**TABLE 4:** Didactic and clinical components for extracorporeal shockwave therapy (ESWT) core curriculum

ESWT core curriculum components	
Didactic	
Lectures	Principles of ESWT: Introduction to physics, knobology, safety protocols/techniques, ergonomics, informed consent (risks, benefits, side effects), time‐out protocols, and basic ESWT applications
Hands‐on demonstration sessions	Led by senior residents, fellows, and/or attendings
Trainee practice sessions	Direct supervision and feedback from senior residents, fellows, and/or attendings
Clinical	
Rotation orientation	Review of milestones, competencies, and rotation goals
Faculty observation	Preprocedural, procedural, and postprocedural protocols
Progressive trainee performance	Direct faculty supervision with procedural invovlement based on current milestone achievement
Feedback sessions	Both formal sessions at specific time points during rotation (ie, midpoint), as well as informal/immediate feedback during procedure or debriefing session afterward to allow for focused practice aligned with milestone goals
Procedural documentation	Review of necessary elements and/or billing procedure if applicable
Competency assessment	Based on outlined ESWT milestones in Table 8

Proposed ESWT milestones for resident education along with target graduation competencies are presented in Table 5 and range from foundational (level 0) to aspirational (level 5). Aspirational competencies are generally reserved for fellows or practicing clinicians. As a resident progresses through milestones, the complexities of diagnoses and skill required to perform ESWT should increase as well (Table 6). Beginner‐level diagnoses include those that involve treatment of superficial structures (eg, plantar fasciopathy) or large muscle‐tendon units (eg, mid‐substance Achilles tendinopathy). Intermediate‐level diagnoses include muscle‐tendon‐bone units (eg, insertional Achilles tendinopathy, patellar tendinopathy, or proximal hamstring tendinopathy) as treatment on or around bony prominences requires additional care and control of the device, in addition to potential dose titration. Treatment of spasticity can also be considered an intermediate‐level skill. Advanced‐level procedures include treatment of bone (eg, bone stress injuries/fractures or delayed/nonunions), joint, and/or periarticular pathologies given higher energy level requirement and associated potential for increased procedural pain and/or effect on nearby anatomic structures. Further, ESWT treatments that require ultrasound experience for targeted application (eg, calcific tendinopathies) or those with nearby sensitive neurovascular, reproductive, or pulmonary anatomy should be reserved for advanced practice.

**TABLE 5:** Sample extracorporeal shockwave therapy (ESWT) milestones for trainee education with associated target competencies

Procedural skills: Extracorporeal shockwave therapy					
Level 0	Level 1	Level 2	Level 3	Level 4 (Graduation target)	Level 5 (Aspirational)
Foundational understanding of ESWT principles: ‐Physics ‐Knobology ‐Ergonomics ‐Safety protocols	Clinical knowledge of ESWT application: ‐Indications for use (radial vs. focused) ‐Risks ‐Benefits ‐Side effects ‐Contraindications	Demonstration or verbal description of appropriate protocols in a simulated setting: Preprocedural: ‐Setup ‐Positioning & ergonomics ‐Informed consent Procedural: ‐Device placement & settings ‐Titration goal ‐Anatomic landmarks, etc. Postprocedural: ‐Patient counseling ‐Device cleaning Performance of informed consent and time‐out in a clinical setting Performance of beginner level ESWT procedures with **supervision** + signficant hands‐on assistance	Performance of beginner/intermediate level ESWT procedures with supervision + little to no hands‐on assistance (may require verbal assistance) Ability to titrate dose based on clinical response Generates appropriate documentation	Performance of advanced level ESWT procedures with supervision + little to no assistance Experience across an expanding spectrum of diagnoses and patient‐specific factors, requiring individualization of care Ability to suggest protocol modifications based on clinical response Teaching of peers Publication of peer‐reviewed work related to ESWT with significant faculty mentorship	Performance of **wide** range of advanced level ESWT procedures adeptly and efficiently Independent modification of treatment protocols to achieve targeted and measurable patient outcomes with integration of current literature and evidence‐based treatment plans Teaching of peers and faculty Independent publication of peer‐reviewed work related to ESWT
□ □ □ □ □ □ □ □ □ □ □ Selection of a box between levels indicates that some, but not all, milestones in the higher level have been achieved.					
Comments:					

**TABLE 6:** Proposed progression of clinical teaching based on trainee experience and procedural complexity

Procedural experience	Appropriate clinical teaching with example diagnoses
Beginner	Superficial soft tissue structures Plantar fasciopathy Large muscle‐tendon units (without inclusion of adjacent bone) Achilles tendinopathy (noninsertional)
Intermediate	Muscle‐tendon‐bone units (with inclusion of adjacent bony origin or insertion) Achilles tendinopathy (insertional) Patellar tendinopathy Proximal hamstring tendinopathy Spasticity
Advanced	Bone stress injury/stress fracture Joints/periarticular structures Tibiotalar, subtalar joints Arthropathy‐related marrow edema Avascular necrosis Structures that require ultrasound expertise for targeted treatment Calcific tendinopathy Structures adjacent to key neurovascular, reproductive, or other anatomy including lung parenchyma Osteitis pubis Spine/axial indications

Barriers to incorporation of ESWT into residency curriculum likely mirror those for other procedures. The most significant barriers to teaching musculoskeletal ultrasound as identified by PM&R residency program directors include inadequate knowledge and experience of preceptors, lack of equipment availability, and inadequate literature defining what should be taught to PM&R residents.^29^ Accordingly, consolidation of ESWT training into a single rotation with higher volume exposure may be the most feasible way to overcome these logistical barriers given likely reduced access to preceptors and ESWT equipment compared to both ultrasound‐ and fluoroscopy‐guided procedures. Before incorporation of an ESWT curriculum for trainees, programs should assess the resources that are available and adjust target volumes, milestones, and competencies appropriately. Importantly, lack of PM&R resident interest was not identified as a barrier to teaching musculoskeletal ultrasound in the aforementioned study.^29^ Implementation of an ESWT curriculum will allow residency and fellowship training programs to further fulfill an identified educational objective for trainees.

### Ergonomics

**FIGURE 1:**
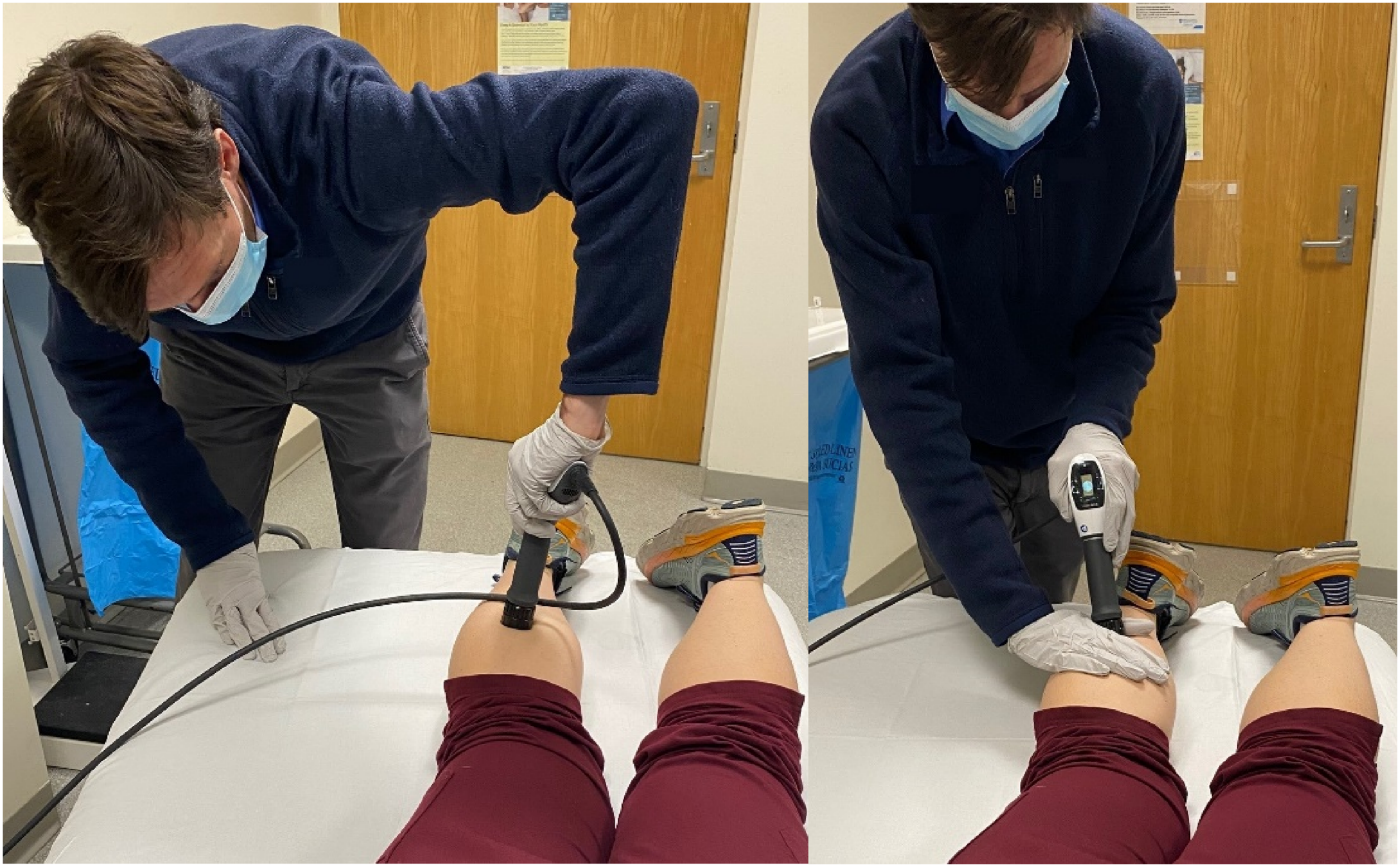
Left photograph with poor ergonomic positioning. Right photograph with proper ergonomic positioning

Similar to other procedures, appropriate setup and ergonomics should be used when performing ESWT. The table height should be adjusted to allow clinicians to perform the treatment with their shoulder and spine in neutral and to leverage gravity. The machine's screen should always be in view. It is important not to “back‐hand” the device during treatment and avoid unnecessary rotatory stress in body position (Figure 1). Like ultrasound, ESWT is a “contact sport” that requires the clinician to maintain direct contact with the patient (similar to proper use of transducer with ultrasound technique) to help direct the treatment. Adjustments are made during treatments to ensure principles of clinical focusing are employed (identifying all sites of pain for a given condition). With radial shockwave devices, avoid kinking the air pressure hose or turning on the device without contact with a patient to avoid damaging the unit.

### Use of technician

Delivery of shockwave can incorporate the use of technicians who serve as valued members of the treatment team and require proper training to administer treatment. Initial education should include shadowing and observing patient visits, understanding the principles and correct settings in use of focused and radial shockwave devices, and practicing correct documentation and cleaning procedures. Proper technique must be used in order for the treatment to be fully effective, therefore applying light, moderate, or firm amount of pressure to the area of injury as instructed by the physician while also holding the devices in an ergonomically efficient manner. The technician should review the patient's previous note as guidance for their current visit and execute the same procedure as directed by the physician. Also, teaching appropriate bedside manner will help reduce patient anxiety and encourage patient‐to‐technician communication throughout the procedure. Frequently asked questions are helpful to learn and be able to address while staying within scope of practice (Table 7).

**TABLE 7:** Summary of shockwave procedure and postprocedure guidelines

Procedure Guidelines	Shockwave dose Start with low energy levels, titrate up as tolerated. Treatment Use of analgesia may reduce ability to use clinical focusing technique. Treatment timeline Generally 3‐5 sessions with the exception of treatment for bone and spasticity at 1 week intervals.
Post‐Procedure Guidelines	Medications/modalities Avoidance of nonsteroidal anti‐inflammatory drugs, ice, fluoroquinolones, and corticosteroids. Acetaminophen can be used for pain relief. Physical therapy Couple with ESWT to restore tissue function and potentially optimize treatment response. Return to activity Lack of post‐procedure immobilization except in selective cases Activity limitation

## COUNSELING PATIENTS

### Potential risks and side effects

In the hands of a qualified physician, ESWT is generally a safe and well‐tolerated procedure. Common complications or adverse effects are described in Table 8.^1^, ^6^, ^14^ Although ESWT is considered a relatively low‐risk procedure, there have been a few cases with major complications reported in the literature. Across all published randomized control trials, Costa et al. reported two cases of Achilles tendon rupture within 2 weeks of initial shockwave treatment with F‐SWT in women older than 60 years of age.^30^

**TABLE 8:** Potential risks and side effects of extracorporeal shockwave therapy

Local effects	Pain at applicator site
	Skin erythema
	Skin bruising
	Hematoma formation
	Nerve irritation with numbness or tingling
	Superficial edema
Systemic effects	Headache
	Migraine

Pain during the treatment is expected as goals are to identify sites of pain.^23^ Hyperstimulation analgesia may explain why a majority of patients will have relief in pain following the treatment. The duration of postprocedure pain relief has not been conclusively shown to predict treatment response. Some patients may experience increased pain in the days following treatment and should be encouraged to use acetaminophen (provided that there are no contraindications) or other topical analgesia as needed. In general, avoiding use of nonsteroidal anti‐inflammatory drugs (NSAIDs) for breakthrough pain is advised as key aspects of the inflammatory cascade may contribute to tissue healing.^5^

### Activity

Level of physical activity following ESWT requires clinical decision making based on factors including type of shockwave treatment, functional status, and type of condition treated. Protocols on structured activity for a given condition are outside the scope of this report. Bone pathology requires activities to be modified to achieve pain‐free status to allow for bone healing, including consideration of crutches or walking boot. Patients receiving treatment of tendons, soft tissue conditions, and spasticity can maintain some level of physical activity but should follow published guidelines for load management when available for a given condition (eg, Achilles tendinopathy).^31^ Those with advanced tendon or soft tissue injuries (eg, partial tear of Achilles tendon) and older adults (above 60 years) should be counseled on limited available data for possible elevated risk for tendon rupture reported in the first 2 weeks of treatment with high‐energy F‐SWT,^30^ and they should be advised to modify activity in these cases. All patients should be counseled that hyperstimulation analgesia immediately following treatment may reduce pain and activity should not be advanced during this time. Overall load management to keep pain at a minimum is advised across all conditions treated and to ensure physical therapy gains are met.

### Contraindications to shockwave therapy

The International Society for Medical Shockwave Treatment (ISMST), established in September 1997, has compiled a list of contraindications to ESWT (May 2019 version).^14^ Table 9 lists contraindications to ESWT as recommended by the ISMST. There is strong evidence to avoid ESWT in the cases of active infection (osteomyelitis), pregnancy, or adjacency to sites of known cancer. Additionally, high‐energy ESWT treatments should not be directed at lung tissue, brain or nerves (spine), epiphyseal plate/sites of skeletal immaturity, and in cases of severe coagulopathy.^1^ Guidelines for use of ESWT may change, as chronic infection and a cancer diagnosis are no longer considered contraindications.^1^, ^14^

**TABLE 9:** Contraindications to extracorporeal shockwave therapy

Absolute contraindications (all energy treatments)	Active infection (ie, osteomyelitis)
	Malignant tumor (focused shockwave)
	Pregnancy
Relative contraindications (high‐energy treatments)	Brain or nerve in treatment focus
	Lung or pleura in treatment focus
	Significant coagulopathy
	Epiphyseal plate in treatment focus
Important considerations	Cardiac pacemakers or other implantable devices
	Current nonsteroidal anti‐inflammatory druguse
	Current anticoagulation use
	Recent corticosteroid injections

Clinical factors to consider in the decision to provide ESWT include current NSAID and/or anticoagulation use, recent corticosteroid injections, and the presence of cardiac pacemakers or devices. The safety of lithotripsy in patients with pacemakers or implantable cardiac devices has been studied with continuous monitoring via telemetry during the shockwave therapy, immediate interrogation of the device following treatment, and termination of the treatment should any arrhythmias arise.^32^ Regarding anticoagulation use or clotting disorders, ESWT should be used judiciously in these instances, given that one of the common adverse effects is hematoma formation.^14^ Coagulopathy is cited as a contraindication in the ISMST 2019 guidelines, although specific mention of blood thinning medications (NSAIDs, warfarin) is not included. Often, these factors exist as exclusion criteria and may be cited as contraindications to treatment by ESWT device manufacturers. As such, it is difficult to ascertain the safety of ESWT in these cases as they are not included in research studies.^25^

## FUTURE RESEARCH

Future research should focus on developing standardized techniques and treatment protocols for specific diagnoses. Validated patient‐reported outcome measures including both pain and functional components should be used in clinical practice to evaluate treatment outcomes for patients. Further high‐level evidence is needed to better compare the effects of radial, focused, or combined ESWT versus sham or placebo ESWT for specific refractory musculoskeletal and neurologic diagnoses. Additionally, the use of ESWT alone or in combination with orthobiologic injections is a specific area of interest that requires additional research. From a clinical education perspective, further development and implementation of ESWT curriculum into residency and fellowship programs will allow for enhanced procedural exposure and training.

## DISCLOSURES

Dr Tenforde reports support from Uniform Health Services for financial support to conduct research on photobiomodulation and shockwave for management of Achilles tendinopathy and DJO Global for LiteCure device to conduct study photobiomodulation and shockwave for management of Achilles tendinopathy
